# The connection between the primary care and the physical activity sector: professionals’ perceptions

**DOI:** 10.1186/s12889-016-3665-x

**Published:** 2016-09-21

**Authors:** Karlijn E. F. Leenaars, Annemiek M. E. Florisson, Eva Smit, Annemarie Wagemakers, Gerard R. M. Molleman, Maria A. Koelen

**Affiliations:** 1 Wageningen University & Research Centre, Department of Social Sciences, Health and Society Group, P.O. Box 8130, EW Wageningen, The Netherlands; 2 Academic Collaborative Centre AMPHI, Primary and Community Care, Radboud University Medical Center, P.O. Box 9101, 6500 HB Nijmegen, The Netherlands

**Keywords:** Intersectoral collaboration, Primary health care, PA sector, Broker role, Qualitative study

## Abstract

**Background:**

To stimulate physical activity (PA) and guide primary care patients towards local PA facilities, Care Sport Connectors (CSC), to whom a broker role has been ascribed, were introduced in 2012 in the Netherlands. The aim of this study is to assess perceptions of primary care, welfare, and sport professionals towards the CSC role and the connection between the primary care and the PA sector.

**Methods:**

Nine focus groups were held with primary care, welfare and sport professionals within the CSC network. In these focus groups the CSC role and the connection between the sectors were discussed. Both top-down and bottom-up codes were used to analyse the focus groups.

**Results:**

Professionals ascribed three roles to the CSC: 1) broker role, 2) referral, 3) facilitator. Professionals were enthusiastic about how the current connection was established. However, barriers relating to their own sector were currently hindering the connection: primary care professionals’ lack of time, money and knowledge, and the lack of suitable PA activities and instructors for the target group.

**Conclusions:**

This study provides further insight into the CSC role and the connection between the sectors from the point of view of primary care, welfare, and sport professionals. Professionals found the CSC role promising, but barriers are currently hindering the collaboration between both sectors. More time for the CSC and changes in the way the primary care and PA sector are organized seem to be necessary to overcome the identified barriers and to make a success of the connection.

**Trial registration:**

Dutch Trial register NTR4986. Registered 14 December 2014.

## Background

Intersectoral collaboration – defined as people and organizations from multiple sectors working together for a common purpose – has become an increasingly popular health promotion strategy [1]. Intersectoral collaboration is challenging because it means working in a new area or setting, with new people with different backgrounds, interests, and perspectives [2–4]. A health broker seems to offer the promise of improving intersectoral collaboration [5]. In 2012, the Dutch Ministry of Health, Welfare, and Sport introduced neighbourhood sport coaches (in Dutch *Buurtsportcoach*) – to whom a broker role has been ascribed – to stimulate physical activity (PA) and connect the sport sector with other sectors. Some of these coaches, the so-called Care Sport Connectors (CSCs), are employed specifically to connect the primary care and the PA sector in order to guide primary care patients towards local PA facilities.

Previous studies have shown that connecting the primary care and the PA sector is challenging for various reasons. First, differences between both sectors, such as culture (professional organization versus voluntary organization) and different shared interests (program interest versus increasing member numbers) can hinder collaboration between the primary care and the PA sector [6]. Secondly, factors relating to both sectors can hinder the referral of primary care patients towards local PA facilities. Health professionals consider their lack of time, formal education, competing priorities, and their perception of patients’ lack of motivation to be physically active as factors that negatively influence PA promotion [7–9]. In addition, the lack of suitable PA activities for the target group [10], sport professionals lack of medical knowledge, and lack of feedback on patients’ progress were seen as factors relating to the PA sector that could hinder the referral of primary care patients [6]. CSCs revealed similar perceived barriers in their work to connect both sectors: a lack of time and knowledge of primary care and sport professionals, lack of suitable PA activities, and own interests of primary care professionals [11].

Although experience of professionals is already contributing somewhat to our understanding of the connection between the primary care and the PA sector, to our knowledge this has not been studied in relation to a broker role employed to connect both sectors. In our previous study, in which we described a first impression of the CSC role, it appeared that most CSCs found it hard to establish a structural collaboration because of perceived barriers [11]. The aim of this study is to assess perceptions of primary care, welfare, and sport professionals towards the CSC role and their experiences in the connection between the primary care and the PA sector in order to further optimize this connection. Research questions addressed were: 1) what is the perception of professionals within the CSC network towards the CSC role and 2) what is the perception of these professionals towards the connection between the primary care and the PA sector?

## Methods

This study is part of a larger project in which a multiple case study is being conducted in nine municipalities spread over the Netherlands from 2014 to the end of 2016 to study the CSCs’ role and impact in connecting primary care and the PA sector, and residents’ participation [12].

### Study design

To gain a comprehensive insight into perceptions on the CSC role and the connection between the primary care and the PA sector, we conducted a qualitative study in which focus groups were held within the CSC network.

### Selection and study population

Nine focus groups were held with primary care, sport, and welfare professionals within the network of 10 CSCs. The selection differed for professionals working with the CSC in a partnership and those who worked individually on a project basis with the CSC. In five cases, we conducted a focus group in a meeting of a partnership in which representatives of the municipality, CSC, primary care, welfare, and sport professionals worked together to promote PA within the community. Therefore, in these focus groups, representatives of the municipality were also present. All professionals (*n* = 29) part of the partnerships attended these focus group. In the other four cases, CSCs working on a project basis with the professionals in their network provided the names of 63 professionals. CSCs and professionals were invited by e-mail to participate in the focus group. In total, 24 professionals attended these focus groups, the other invited professionals were due to a lack of time not available to participate in the focus group.

In total, 21 primary care professionals (13 physiotherapists, three GPs, three representatives of a home care organization, one exercise therapist, and one representative of a community health centre), 16 sport professionals (eight representatives of a sport club, six exercise instructors, and two representative of a fitness centre), eight representatives of welfare organizations (four of which offer exercise lessons, and four of which do not), and nine representatives of the municipality attended the focus groups. One professional was both a primary care professional and a sport professional, and therefore part of both groups. In addition, CSCs attended the focus group (*n* = 9) (Table 1).

**Table 1:** Focus group participants

		Primary care professionals			Sport professionals		Welfare professional		Others		
Focus group	Structure of the collaboration	GP	Physio-therapist	Others	Sport club	Others	With PA offer	Without PA offer	Municipality representative	CSC	Total
1.	Partnership	0	0	2	1	0	1	0	2	1	7
2.	Partnership	1	1	0	2	1	0	1	3	1	10
3.	Partnership	1	0	0	1	1	1	1	3	2	10
4.	Partnership	0	1	0	2	1	1	0	1	0*	6
5.	Project basis	1	0	1	0	1	0	0	0	0**	3
6.	Project basis	0	2	0	0	2	1	0	0	1	6
7.	Project basis	0	3	0	0	0	0	0	0	1	4
8.	Project basis	0	3	1	2	1	0	0	0	1	8
9.	Project basis	0	3***	1	0	0	0	2	0	2	8
Total		3	13	5	8	7	3	5	9	9	62

*CSC quit his job and was therefore not available for the focus group

**This municipality does not employ a CSC but appointed existing organisations as a CSC

***One primary care professionals is also an exercise instructor

### Procedure

The focus groups were held between June and October 2015. They took place at the CSCs’ workplace and lasted approximately 1.5 h. Eight focus groups were conducted by two researchers (KL and ES or AF); one focus group was conducted by one researcher (KL). At the beginning of each focus group, professionals were informed about the procedure and signed an informed consent.

The focus group consisted of two parts: the CSC role (part I), and the connection between the primary care and the PA sector (part II). CSCs only participated in the second part of the focus groups. In this way, professionals could speak freely about the CSC role, and in the second part, professionals and the CSC could provide one another with direct feedback to stimulate collaboration between the CSC and the professionals. At the beginning of part I, all professionals completed a form in which they had to prioritize 10 possible CSC tasks, based on the Dutch Knowledge Centre Sport’s CSC competence profile [13], by degree of importance (1 = very important, 10 = not at all important). Then, each participant explained one by one his/her choice of the three most important tasks. Subsequently, a discussion took place about the CSC in their municipality. To facilitate this discussion the following question was asked: *“To what extent is the CSC an added value to you as a primary care, welfare, or sport professional?”.* In part II, the topic was the attitude and expectation towards the connection between the sectors. To facilitate the discussion, all professionals first described their own role and their expectations about other professionals. Subsequently, the connection between the sectors was discussed. Guiding questions were for example: “*How do you evaluate the current connection between the primary care and the PA sector in your neighbourhood/municipality?”* and *“How do you see the connection between the primary care and the PA sector in the course of two years?”.* In one focus group, we performed only part II of the focus group. In this municipality, no CSC was employed, but existing organizations received the CSC funding, and therefore part I was not applicable.

### Data analysis

The focus groups were audiotaped and transcribed. The data analysis was based on Creswell’s [14] six steps for qualitative data analysis. So, after the data were organized and prepared for the analysis, with the participants divided in four groups (primary care, sport, welfare, others) and the focus groups divided in two groups (partnership or project basis), the transcripts were read. Focus groups were divided in these two groups, due to the different starting point. It is possible that professionals of the partnership share a similar vision towards the CSC role and the connection between both sectors because they work together on stimulating PA, while professionals of the other four focus groups did not work together and worked on a individually basis with the CSC. In the third step, the transcripts were coded and analysed using software for qualitative analysis (Atlas.ti). Top-down codes related to the structure of the collaboration and facilitators and barriers in the connection between the primary care and the PA sector were defined based on the results of the interviews with the CSCs [11] and on existing literature on the connection between both sectors [6]. In the fourth step, the codes were clustered into the following themes: the CSC role, the connection between the primary care and the PA sector, and facilitators and barriers relating to the sector and not relating to the sector. During steps five and six, more bottom-up codes were assigned to the various themes, for example in the theme ‘the CSC role’, the new codes identified were: broker role, referral, facilitator (Table 2).

**Table 2:** Code list

Themes	Top-down codes	Bottom-up codes
CSC role	• Attitude	• Broker role
	• Added value	• Referral
		• Facilitator
Collaboration in the connection between the primary care and the PA sector	• Role	• Attitude
	• Partnership	• Expectations
	• Project basis	
Barriers and facilitators relating to sector	• Time and money	• Reimbursement
	• Lack of knowledge of the PA offer	
		• Awareness of the PA offer
	• Own interest	
	• Suitable PA activities	• Competitive position
	• Adequate PA instructors	
Barriers and facilitators not relating to the sector	• Personal perceptions	• Effectiveness
		• Policy
		• Target group

The whole data analysis process was performed independently by two researchers (KL and AF). After step three, the transcripts were discussed until consensus on the assigned codes was reached between the researchers. Also, the researchers discussed the interpretation of the data to reach consensus. The most discussion was held on the theme attitude of the collaboration between the primary care and the PA sector, because some professionals were satisfied while other professionals mostly mentioned points for improvement. Once the data analysis was completed, the results were discussed within the research team.

As our aim was to study the perceptions of primary care, welfare, and sport professionals, citations of others (municipal representatives and CSCs) were not included in the analysis. The municipal representatives were policymakers and consequently involved in policymaking to implement CSCs, but they did not fulfil an active role in the connection between both sectors. CSCs did not have an active role in the focus groups and used the focus groups as a way to explain their work to the professionals. The assignment in part I was analysed by calculating a mean of each of the possible CSC tasks, ranked by degree of importance (1 = very important, 10 = not at all important) (Table 3).

**Table 3:** Results of the assignment in part I of the focus group

	Sport (*n* = 10)	Primary health care (*n* = 20)	Welfare (*n* = 7)
1. Acts like a broker and matcher between the demand determined and the supply realized	5.5 (SD = 4.2)	5.1 (SD = 3.1)	5.7 (SD = 2.8)
2. Takes care of/arranges the inventory of the needs for sport and exercise activities within the work field (s)	3.5 (SD = 1.6)	4.8 (SD = 2.7)	6.3 (SD = 2.8)
3. Creates a network with the parties from the care, sports, and welfare sectors relevant to the target group or links up with existing networks and expands these, if necessary	3.4 (SD = 2.6)	3.8 (SD = 2.3)	3.4 (SD = 2.1)
4. Maps out the range of activities available to the target group and also considers the exercise activities made available by other sectors like welfare, apart from the regular sports and exercise activities provided	5.5 (SD = 2.3)	3.5 (SD = 1.6)	3.7 (SD = 1.3)
5. Helps sports and exercise providers in developing an appropriate range of activities	5 (SD = 2.5)	6.8 (SD = 2.6)	6.7 (SD = 2.3)
6. Organizes and coordinates at the execution level a coherent range of activities in the areas of sports and exercise	6.2 (SD = 2.0)	6.4 (SD = 2.2)	4.7 (SD = 3.3)
7. Acquires/scouts active participants for various activities at the relevant target group’s specific care and welfare organizations	6 (SD = 3.2)	5.8 (SD = 2.9)	7 (SD = 2.2)
8. Guides participants towards sports and exercise activities, in consultation and, if necessary, in collaboration with a care and welfare organization	5.5 (SD = 2.9)	4.4 (SD = 3.1)	3.1 (SD = 2.9)
9. Provides information and arranges for the enhancement of the expertise of trainers and managers at sport-providing organizations	7.9 (SD = 1.7)	8.6 (SD = 1.7)	8.9 (SD = 1.1)
10. Organizes, coordinates, and performs other health promoting activities in the neighbourhood in collaboration with relevant parties from the neighbourhood	6.5 (SD = 3.2)	5.9 (SD = 2.6)	5.4 (SD = 3.2)

*Note:* Mean scores of assignment I in eight focus groups. Tasks were ranked by degree of importance (1 = very important, 10 = not at all important

*Note:* participants of the focus group in municipality 9 did not full in assignment 1 because this municipality did not employed a CSC function, therefore the assignment was not applicable. In addition, 2 sport professionals in 2 other focus groups only prioritized 3 tasks, therefore the results of these assignments were not included

## Results

### Role of the care sport connector

In the focus groups, primary care, welfare, and sport professionals ascribed three roles to the CSC: 1) broker role, 2) referral, and 3) facilitator.

#### Broker role

Almost all professionals, regardless of whether they were already part of a partnership, mentioned that an important task for the CSC was to fulfil the broker role and stimulate collaboration between professionals (task 3, mean = 3.4, mean = 3.8, mean = 3.4). Primary care professionals in particular stressed the importance of a connection between sectors, because more collaboration may result in increased referral of patients.

*“I believe that, right now, to me it is important that the CSC makes it so that the separate domains… that preferably there are no separate domains anymore between care, sports, and welfare. That these come into contact and start using one another’s strength.” (Physiotherapist, #9)*

An important feature of the CSC was to have a bird’s eye view of the whole neighbourhood and to be the driving force and/or initiator in the connection between both sectors.

#### Referral

The referral of primary care patients and residents towards local PA facilities was also highly prioritized as a CSC task, especially by primary care (task 8, mean = 4.4) and welfare professionals (task 8, mean = 3.1), as a way to stimulate PA and increase the health of their patients and residents.

*“Guiding our patients towards appropriate exercise activities so that they will visit me less frequently and also feel better both physically and mentally.” (GP, #2)*

Although in the assignment the referral function was not highly prioritized by sport professionals (task 8, mean = 5.5), in the focus groups they often mentioned it as an important CSC task. They would like to have more participants in their exercise lessons. Identifying the need for PA activities for residents is therefore an important prerequisite.

*“That she also encourages the target group towards exercise and sports and helps people find their way to PA activities more easily.” (Sport instructor, #6)*

#### Facilitator

Providing an insight into the current PA offer was another task that was highly prioritized by primary care (task 4: m = 3.8), and welfare professionals (task 4: m = 3.7). Primary care and welfare professionals mentioned in the focus groups that this is an important task because neither the target group nor the professionals are familiar with all PA activities in the neighbourhood. Therefore, welfare professionals would like to have an insight into existing PA activities for the target group, and primary care professionals would like to know this so that they could refer patients towards these activities.

*“You will then have to properly map out what range is available and apart from regular sports… it would be nice if a professional, if we did not have to do so in our own time, our limited time.” (GP, #5)*

Providing an insight in the needs for PA activities (task 2, m = 3.5), and helping sport professionals in developing new PA activities (task 5: m = 5) were highly prioritized by sport professionals as important tasks. However, sport professionals did not elaborate on this in the focus groups and mostly mentioned providing an insight in existing PA activities as an important task. Especially because according to sport professionals, primary care and welfare professionals are not familiar with the PA offer.

#### Added value of the CSC

In general, professionals in all nine focus groups were positive about the way the CSCs fulfilled their role. The CSC’s personality was often perceived as pleasant. CSCs were enthusiastic, visible, stimulating, approachable, and active.

*“What I believe to be an added value is that she is visible through… that she actually does something… Like, ehm, just as you put it: ‘I am glad, I am glad, for if I call the CSC, some action is taken’.” (Welfare professional, #6)*

Although the CSC role was perceived as positive by all professionals, in four focus groups some professionals mentioned that the CSC was not yet seen as an added value for their organisation. Either because up to now there was not much collaboration in the network or because the results of the CSCs’ work were so far not of sufficient value to these professionals.

*“Well, you can sometimes hear someone say: ‘it is somewhere in the proximity of the [welfare organization] and not visible enough in other areas of the neighbourhood’.” (Welfare professional, #3)*

### Connection between the primary care and the PA sector

The connection between the primary care and the PA sector was differently established in each CSC network. In five CSC networks, a partnership had been established between primary care, sport, and welfare professionals: four partnerships were organized by the municipality, one by a sport organization. Professionals in these partnerships worked together to organize activities to promote PA or implemented a referral scheme. In the other four networks, professionals worked individually on a project basis together with the CSC in the organization of activities to promote PA or in the referral of primary care patients. Because of the differences in the form of collaboration, professionals had different attitudes and expectations about the connection between both sectors (Table 4).

**Table 4:** Results of the professionals’ perceptions of the connection between the primary care and the PA sector

Focus group	Structure of the collaboration	Role professionals	Attitude connection	Expectations of the connection
1.	Partnership organized by the municipality	▪ Part of partnership ▪ Activities to promote PA	▪ Good start ▪ Takes time ▪ Not a clear mission in the steering group ▪ Not many concrete actions	▪ Other organizations should be involved in the partnerships ▪ Continuity of CSC funding and the steering group ▪ More contact with one another
2.	Partnership organized by the municipality	▪ Part of partnership ▪ Referral scheme	▪ Good start, in which the CSC is indispensable ▪ Clear shared vision about the CSC	▪ More collaboration between professionals ▪ Referral should be a matter of course ▪ Continuity of CSC funding and the steering group
3.	Partnership organized by the municipality	▪ Part of partnership ▪ Sport consultation at community health centre, coordination of the PA offer at community centre	▪ Partially positive. Much has been achieved but there is room for improvement ▪ The connection takes time ▪ No shared mission because of different interests	▪ More collaboration with other organizations ▪ More time is needed
4.	Partnership organized by the municipality	▪ Part of partnership ▪ Activities to promote PA	▪ Partnership is an added value because they know one another and development of activities ▪ Clear and shared mission	▪ More organizations involved in the partnership ▪ Create more publicity for the work of the partnership
5.	Partnership organized by a sport organization	▪ Part of partnership ▪ Activities to promote PA	▪ The partnership is valuable because professionals know one another	▪ CSC should be responsible for the collaboration and the connection ▪ Professionals are willing to help with the implementation of activities
6.	Project basis	▪ Organization of fit tests ▪ Referral	▪ Promising: good start to a first collaboration between professionals ▪ The referral of patients is getting better but is still difficult	▪ Regular meetings with all partners ▪ Referral should be a matter of course
7.	Project basis	▪ Organization of activities to promote PA and referral	▪ Added value ▪ Hard to refer and guide patients towards local PA facilities	▪ Regular meetings with all partners so that professionals can meet one another ▪ Referral should be a matter of course
8.	Project basis	▪ Organization of fit tests ▪ Sporadic referral	▪ The connection is difficult because of unfamiliarity with one another ▪ Too passive	▪ A clear referral scheme ▪ Regular meetings with all partners so professionals can meet one another
9.	Project basis	▪ Referral ▪ Organization of PA activities	▪ Promising start: further development necessary ▪ Takes time	▪ Involve more organizations in the connection ▪ The connection should be a matter of course ▪ Regular meetings with all partners so that professionals can meet one another

#### Partnership: attitude and expectations about the connection between both sectors

Professionals who belonged to a partnership mentioned that they were enthusiastic about the partnership and that it was a good first step towards more collaboration between the sectors. Professionals in three partnerships mentioned a shared mission, which facilitated the collaboration.

*“I now have seven people around the table whom I can deploy now. ‘Hey, I have seen you around somewhere, couldn’t we have some time?’ That is a joint part, isn’t it? We also have a joint goal and we also have, well, joint interests.” (Sport professional, #4)*

However, two partnerships had not yet achieved the desired results, and more time and attention was needed to further improve the collaboration between the sectors. In these partnerships, professionals did not have a clear shared mission on the CSC role and the connection between the sectors.

*“But I do hear a number of different points of departure here; we have different points of departure. For instance, […] says emphatically that there is a shortage in the range available and I hear others say ‘the range is adequate, there is quite a lot on offer, but there, it doesn’t get here.’ There are, there are a number of things about which we simply reason from different assumptions; that makes it rather…” (GP, #3)*

The professionals expect to expand the current connection, to involve more partners, and the referral of primary care patients should be a matter of course for organizations. More time and continuity of the CSC role is therefore needed.

#### Project basis: attitude and expectations about the connection between both sectors

Professionals in networks working on a project basis with the CSC were also enthusiastic about how the connection between both sectors was established. The organization of an activity was seen as a good way to stimulate collaboration between the professionals.

*“A collaboration is simply very difficult if you start from scratch. Everyone needs to be brought together first, with a goal that involves all the sectors.” (Physiotherapist, #6)*

In all four networks, professionals mentioned that there was a good foundation for collaboration between the sectors. The current form of collaboration was therefore promising, because professionals got to know one another, and the gap between the primary care and the PA sector was reduced. However, more time was needed to further develop the connection, especially because of their unfamiliarity with one another.

*“… We simply just are in too little contact with one another, for you just don’t really know what we… You just don’t really know what we do exactly.” (Sport professional, #8)*

The professionals expected to have a more structural form of collaboration, with regular contact with other professionals, a clear referral scheme, and more involvement of other organizations. The CSC should take the lead in this.

### Perceived barriers and facilitators in the connection between the primary care and the PA sector

In the connection between the primary care and the PA sector two sets of facilitators and barriers were identified: facilitators and barriers relating to the professionals’ own sector, and facilitators and barriers not relating to a sector. Perceived facilitators and barriers did not differ for professionals who worked together in a partnership or who collaborate on project basis.

#### Facilitators and barriers relating to the sector

##### Primary care

In relation to primary care, primary care professionals mentioned: time and money, knowledge about the PA offer, and their own interest as facilitators or barriers in the connection between the primary care and the PA sector. These factors were also mentioned by sport and welfare professionals as their explanation for the lack of involvement of primary care professionals.

##### *Time and money*

Lack of time and money was often mentioned as a barrier to participation, as also the lack of referral by primary care professionals. In addition, the lack of remuneration in the current health insurance system for preventive work was often mentioned as a barrier for the participation of physiotherapists.

*“We have had this and done so, but we haven’t followed up on it, but that is also where part of the problem lies, this, this picture sketched by [participant], we are extremely busy and everything we do here, sitting here, is free. This is my own time; I do not get paid for it.” (Physiotherapist, #7)*

##### *Lack of knowledge about the PA offer*

A lack of knowledge about the PA offer made it hard for primary care professionals to refer patients. They refer patients towards the PA offer with which they are familiar.

*“And you can only refer towards PA activities you know of. For example, I know there are some walking groups on different levels in [neighbourhood], so sometimes I refer patients towards them. But where to I don’t know. They have to, then I say: ‘you have to look it up yourself’.” (Physiotherapist, #8)*

Some primary care professionals also mentioned unfamiliarity with the CSC role as a barrier in the referral of patients.

*“Yes, time, and it is as yet unclear to me as to what the steps are. I would call [name CSS] for the people with a disability, and [name other CSC] for the elderly, I would know whom to call, but I don’t know how things go from there on. It should actually be clearer how ehm.” (Physiotherapist, #9)*

##### *Own interest*

According to primary care professionals, especially physiotherapists, their own interests can contribute to whether or not they participate in the CSC’s work. Some physiotherapists have their own PA offer, and therefore they guide patients towards that.

##### PA sector

In relation to the PA sector, sport professionals mentioned awareness of the PA offer, suitable PA activities, and adequate sport instructors as a barrier or facilitator for the connection between the sectors.

##### *Awareness of the PA offer*

According to sport professionals, there is plenty of PA on offer in the neighbourhood, but this sport offer is insufficiently known by other organizations, mostly because sport professionals do not publicize their PA offer enough.

*“Well, what I believe is important is that the providers, in any field, that they are not known well enough. So, the promotion of those providers is very important, I think. Wherever. A touch of PR.” (Sport professional, #8)*

##### *Suitable PA activities and adequate instructors*

Primary care and welfare professionals mentioned that the PA offer is not always suitable for the target group. The level at sport clubs is too high for the target group, and there are not enough adequate PA instructors. In particular, volunteers working at the sport club are not adequately trained. This was confirmed by the sport professionals, and therefore having enough adequate PA instructors was mentioned as an important facilitator in the connection between the sectors.

*“Make sure that you have good teachers, that you have groups, and that you bring people in. That’s all.” (Sport professionals, #6)*

#### Welfare

Welfare professionals and sport professionals commented on their possible position as rivals: some welfare organizations also offer sport activities and therefore they focus more on their own activities.

#### Facilitators and barriers not relating to the sector

Facilitators and barriers not relating to the sector were also identified: personal perception, effectiveness, policy of the municipality, and the target group.

##### Personal perception

All professionals had an interest in PA and a belief in PA promotion as a means of stimulating a healthy lifestyle, and these were often mentioned as reasons to participate.

*“Have always practiced a lot of sports, still do actively and passively, and so I am very interested in PA sport in our health centre, so I have been aware of the importance of PA for quite some time.” (GP, #5)*

##### Effectiveness

The use of effective projects or activities, according to the professionals, was important for collaboration with the work of the CSC. Showing results and successes can lead to more involvement by professionals and organizations.

*“I say, especially these Exercise Buddies; that really is a fantastic project in my view. That is truly fantastic, for it gets people out of their isolation. Yes, they dare do far more.” (Physiotherapist, #7)*

##### Policy of the municipality

Municipal policy was perceived by the professionals as a facilitator when the policy was supportive of organizations participating in the connection. For example, the current decentralization policy provides an opportunity for primary care professionals to work more preventively, and funding has supported welfare and sport organizations to create PA activities. However, discontinuation of funding was mentioned as a barrier.

##### Target group

The professionals perceived the target group themselves as a barrier in this connection. According to the primary care and welfare professionals, the target group is hard to stimulate, and sport professionals mentioned problems with keeping the target group motivated to continue with the PA activities.

*“Yes, I have also noticed this. People who are not used to exercise very soon find reasons to quit. So, some of the participants that he acquired simply stayed away again.” (Sport professional, #2)*

## Discussion

The aim of this study was to explore the perceptions of primary care, welfare, and sport professionals on the CSC role and the connection between the primary care and the PA sector. Primary care, welfare, and sport professionals ascribed three roles to the CSC: 1) broker role, 2) referral, 3) facilitator. No major differences were identified between the different professionals in their perceptions on the CSC role. The roles that professionals ascribed to the CSC were similar with the roles CSCs fulfilled [11]. Professionals found the CSC role and the current connection promising. However, professionals working on a project basis would like to have more contact with one another, and professionals who belong to a partnership would like to expand the connection towards other organizations.

Prior to the study, it was expected that differences between both sectors would hinder collaboration, especially because a review of the literature showed that collaboration between the primary care and the sport sector was hindered by differences in culture and interests in both sectors [6]. However, these barriers were not identified in this study. Professionals in this study found the connection between both sectors promising, but factors relating to their own sector were currently perceived to hinder this connection. This probably has to do with the form in which the professionals are collaborating. In most cases, the form of collaboration can be characterized as multidisciplinary, whereby different disciplines work independently on different aspects of a project [15]. Therefore, facilitators and barriers relating to their joint services were identified instead of barriers relating to differences between the sectors.

The identified barriers relating to their own sector have also been identified in other studies: lack of time [16–31], lack of remuneration [16, 17, 23, 24, 31], lack of priority [20, 21, 23, 29, 30], and lack of knowledge about the PA offer [18–30, 22, 26, 30] were mentioned as barriers in PA promotion by primary care professionals. In addition, a study of Ooms et al. [10] showed that PA activities at sport clubs in the Netherlands were not suitable for the target group. What is remarkable is that, in spite of the introduction of the CSC function, and especially because this study’s population consisted of professionals who were enthusiastic and willing to participate in this connection, the perceived barriers remain the same and CSCs have not yet succeeded in overcoming them. It is possible that in course of time the CSC will manage to overcome these barriers. Especially because the CSC is a new function and building collaboration structures takes time [32, 33]. Time for the CSC is therefore needed to continue to work on overcoming the perceived barriers and establishing a connection between both sectors. However, the question is also whether the CSC is single-handedly capable of, and responsible for, overcoming these barriers.

The results of this study are therefore relevant for policymakers, municipalities, and organizations working on a connection between the primary care and the PA sector, and can be helpful to further improve the connection. This is particularly so because some of the identified barriers relate to the system in which CSCs and professionals are working. Changes are therefore needed at the system level, because not only a CSC can influence this. For example, the current insurance system in the Netherlands, which only reimburses primary care professionals for their curative treatments, hinder these professionals from participating in projects aimed at prevention or health promotion. Nevertheless, insights from this study are also relevant for CSCs because some of the identified barriers can be influenced by CSCs, and they can start by eliminating these barriers. For example, CSCs could provide an insight for primary care professionals into all PA activities in the municipality, or support sport clubs and their trainers in working with a new target group. More research is necessary to study perceptions of primary care, sport, and welfare professionals about possible strategies to overcome current barriers hindering the collaboration between the primary care and the PA sector.

### Study’s strength and limitations

Some limitations need to be taken into account when these results are being interpreted. Although the aim of this study was to study the perceptions of primary care, welfare, and sport professionals, other professionals were also present as we invited CSCs to attend the second part of the focus groups, and some focus groups took place at a meeting of an existing partnership organized by the municipality. The perceptions of these professionals were not included in the analysis but may have affected the results of the focus groups. However, focus group participants first had to articulate their own opinion as a result of the assignments in part I and part II, thus ensuring that the influence of municipal representatives was limited.

It is possible that the population of this study consisted of professionals with a more positive attitude towards the CSC role and the connection between both sectors. This could have resulted in more positive results. Nevertheless, in the focus groups, professionals still critically discussed the CSC role and the connection between both sectors. In addition, the discussion helped professionals to gain insight into perceived barriers of professionals working in other sectors, which might be a constructive way to support collaboration, overcome some of the barriers and thus, connecting sectors. In some focus groups not all sectors were represented due to cancellations. As a result professionals of these sectors could not elucidate on the comments made by professionals of other sectors. However, it did not affect the results of this study because we discussed perceived barriers of professionals in the connection between the primary care and the PA sector. In addition, there was a limited number of GPs and practice nurses who participated in this study. Their involvement is considered to be important in the connection between both sectors and therefore also their perception on the connection between both sector. However, not in all CSCs networks a GP or practice nurse were present [11]. Therefore, only a small number of GPs and practice nurses could be invited to participate in this study. Only the GPs part of a partnership attended the focus groups. In these cases the focus group took place during an meeting of the partnership.

The roles that primary care, sport, and welfare professionals ascribed to the CSC could have been influenced by the assignment in part I of the focus groups. However, professionals were asked to explain their choices and in the assignment were able to give their own interpretation of the tasks. In addition, the scores were sometimes different than the explanation given by the professionals in the focus groups. For example, almost all sport professionals mentioned that it was important for them to get participants for their PA activities, but they did not highly prioritize this as a role for the CSC. Using both the results meant that a good representation of their perceptions of the CSC role was provided.

## Conclusion

This study provides further insight into the CSC role and the connection between the primary care and the PA sector from the point of view of primary care, welfare, and sport professionals. Although professionals found the CSC function and the current connection between the sectors promising, barriers related to the sectors are at this moment hindering the connection between the primary care and the PA sector. Time must be given to the CSC to further improve the connection and overcome some of the barriers. Changes in the way the primary care and the PA sector are organized seem to be necessary to overcome some of the identified barriers and to make a success of the connection.

## Abbreviations

CSC: Care sport connector
PA: Physical activity
